# Social stress induces autoimmune responses against the brain

**DOI:** 10.1073/pnas.2305778120

**Published:** 2023-11-27

**Authors:** Yusuke Shimo, Flurin Cathomas, Hsiao-yun Lin, Kenny L. Chan, Lyonna F. Parise, Long Li, Carmen Ferrer-Pérez, Samer Muhareb, Sara Costi, James W. Murrough, Scott J. Russo

**Affiliations:** ^a^Nash Family Department of Neuroscience, Icahn School of Medicine at Mount Sinai, New York, NY 10029; ^b^The Brain-Body Research Center of the Friedman Brain Institute, Icahn School of Medicine at Mount Sinai, New York, NY 10029; ^c^Depression and Anxiety Center for Discovery and Treatment, Department of Psychiatry, Icahn School of Medicine of Mount Sinai, New York, NY 10029

**Keywords:** stress, depression, blood-brain barrier, lymphocytes, adaptive immunity

## Abstract

Clinical studies have revealed a high comorbidity between autoimmune diseases and psychiatric disorders, including major depressive disorder (MDD). However, the mechanisms connecting autoimmunity and depression remain unclear. Here, we aim to identify the processes by which stress impacts the adaptive immune system and the implications of such responses to depression. To examine this relationship, we analyzed antibody responses and autoimmunity in the chronic social defeat stress (CSDS) model in mice, and in clinical samples from patients with MDD. We show that socially stressed mice have elevated serum antibody concentrations. We also confirm that social stress leads to the expansion of specific T and B cell populations within the cervical lymph nodes, where brain-derived antigens are preferentially delivered. Sera from stress-susceptible (SUS) mice exhibited high reactivity against brain tissue, and brain-reactive immunoglobulin G (IgG) antibody levels positively correlated with social avoidance behavior. IgG antibody concentrations in the brain were significantly higher in SUS mice than in unstressed mice, and positively correlated with social avoidance. Similarly, in humans, increased peripheral levels of brain-reactive IgG antibodies were associated with increased anhedonia. In vivo assessment of IgG antibodies showed they largely accumulate around blood vessels in the brain only in SUS mice. B cell-depleted mice exhibited stress resilience following CSDS, confirming the contribution of antibody-producing cells to social avoidance behavior. This study provides mechanistic insights connecting stress-induced autoimmune reactions against the brain and stress susceptibility. Therapeutic strategies targeting autoimmune responses might aid in the treatment of patients with MDD featuring immune abnormalities.

## Significance

Patients with stress-related disorders, including depression, show high prevalence rates for autoimmune diseases. To explore the relationship between stress and autoimmunity, we analyzed blood and brain samples from socially stressed mice and patients with major depressive disorder. Stress-susceptible mice showed elevated serum antibody levels and induction of antibody responses in brain-draining lymph nodes. Brain-reactive antibodies were produced in stressed mice and antibody concentrations in the brain correlated with depression-like behavior. Similarly, brain-reactive antibody levels in clinical sera were associated with anhedonia severity. Depletion of antibody-producing cells from mice resulted in stress resilience, confirming the contribution of antibody responses to stress susceptibility. Mechanistic insights connecting stress-induced autoimmune responses against the brain may offer therapeutic strategies for stress-related disorders.

Major depressive disorder (MDD) affects 6% of adults worldwide every year (1, 2). Despite the availability of effective antidepressants and psychotherapies, more than one-third of patients with MDD are resistant to these treatments (3, 4). Such poor treatment outcomes can be ascribed to the heterogeneity of patients with MDD and an incomplete understanding of causal mechanisms responsible for MDD symptoms. Recent reports have revealed that immune abnormalities can be detected in subpopulations of patients with MDD (5–9). Under physiological conditions, the immune system protects against infection and eliminates foreign substances via sequential and coordinated responses called innate and adaptive immunity (10). Innate immune responses are mediated by leukocyte populations, such as monocytes, granulocytes, and dendritic cells, which rapidly and nonspecifically react to pathogens, and eliminate them via several mechanisms, including induction of inflammation.

Stress, a major risk factor for depression, induces inflammatory processes through activation of the innate immune system (11), which has been associated with depression in humans (12), and in mouse models (13–17). Chronic social defeat stress (CSDS) in mice induces behavioral abnormalities that partly resemble clinical symptoms of depression. In the CSDS model, stress-susceptible (SUS) mice show social avoidance, whereas resilient (RES) mice are devoid of such behavioral abnormalities (18). We have previously observed increased levels of circulating innate immune cells (i.e., monocytes and neutrophils), elevated levels of the proinflammatory cytokine interleukin-6 (IL-6), and blood-brain barrier (BBB) dysfunction in the CSDS model and in patients with MDD (19, 20). In particular, we and others have found evidence of increased BBB permeability in the nucleus accumbens (NAc), prefrontal cortex (PFC), and hippocampus (HIP), brain regions implicated in stress and depression (21), in SUS mice following social defeat as well as in MDD subjects (20, 22). While studies investigating inflammatory mechanisms of depression largely focus on the innate immune system, several reports suggest the involvement of the adaptive immune system in neurobehavioral disorders (23–25), but the specific contributions of adaptive immune abnormalities to depression remain unclear.

Adaptive immune responses, mediated by two major populations of lymphocytes, T and B cells, react in a slow but specific manner. One of the most important functions of the adaptive immune response is the production of antigen-specific antibodies from B cells. Adaptive immune cell-dependent antibody responses are primarily induced in organized structures in secondary lymphoid organs called germinal centers. Follicular helper T cells (Tfh), which promote B cell differentiation and proliferation, germinal center B cells (GCB), and plasma cells (PC), the latter of which are responsible for producing antibodies, are all involved in the germinal center reaction, which serves as the basis of humoral immunity against foreign pathogens (26). Tissue-derived antigens are preferentially delivered to specific lymphoid organs, such as the lymph nodes and spleen (SPL). For example, gut-associated lymphoid tissues, such as mesenteric lymph nodes (mLN) and Peyer’s patches, are sites where gut immune responses are induced. The SPL captures blood-derived antigens, and splenic immune cells are activated in mouse stress models which induce depression-like behaviors (27, 28). Cervical lymph nodes (cLN) have been identified as sites of antigen delivery from the brain via lymphatic vessels in the meninges (29, 30).

Clinical studies have shown a high comorbidity between psychiatric and autoimmune disorders (31). Autoimmune reactions against the brain have been implicated in the pathogenesis of psychiatric symptoms such as psychosis and cognitive impairment (32, 33). Several recent studies of autoimmune diseases with comorbid psychiatric symptoms suggest that certain autoantibodies target proteins expressed in the brain and regulate neurobehavioral abnormalities (32, 34–37). However, the role of adaptive immune system dysfunction, including autoimmunity, in the pathogenesis of depression remains unclear. To understand the link between adaptive immune abnormalities, stress, and depression, we analyzed adaptive immune processes in the CSDS model and clinical samples from patients with MDD. We investigated the effects of social stress on immune cell populations controlling antibody production in lymphoid organs such as lymph nodes and the SPL, as well as circulating levels of IgG, the most common type of antibody found in circulation. We next explored whether brain-reactive antibodies are found in sera from stressed mice or patients with MDD, and if antibody levels in the brain are increased in stressed mice. B cell depletion therapies, such as anti-CD20 antibodies, rituximab, ocrelizumab, and ofatumumab, which have been tested in clinical trials or approved for the treatment of some autoimmune diseases (38), were investigated to establish a contributory link between antibody-producing cells and stress susceptibility in the CSDS model.

We found elevated serum antibody levels and strong induction of antibody responses in the brain-draining lymph nodes from SUS mice. Brain-reactive antibodies were induced by stress and correlated with social avoidance behavior, suggesting that autoimmune responses against the brain contribute to stress susceptibility. We observed a similar association between high brain-reactivity of sera and anhedonia in humans, highlighting the clinical relevance of our findings.

## Results

### Social Stress Induces Antibody Production.

**Fig. 1.**
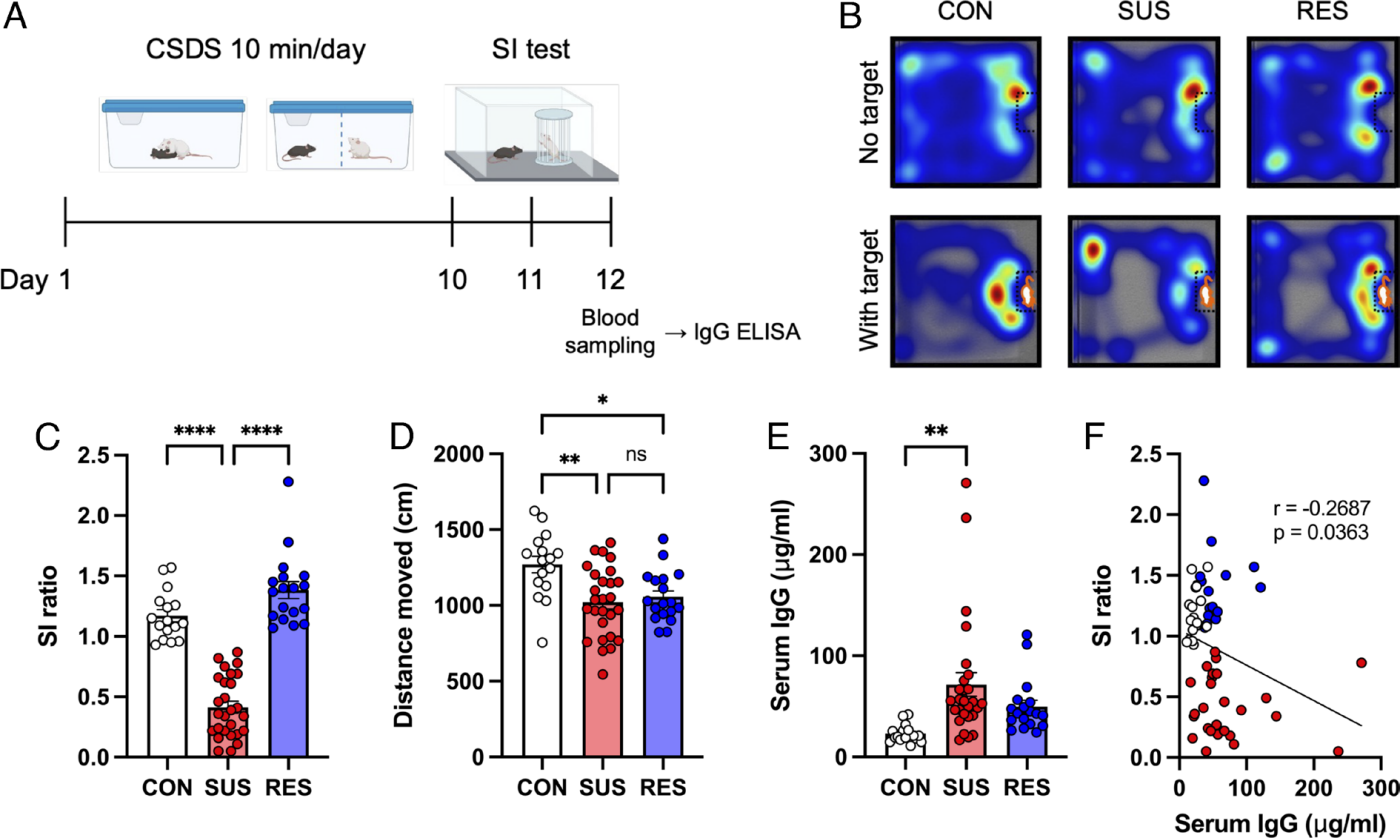
Increased antibody production in SUS mice. (*A*) Experimental outline: Ten days of CSDS followed by a SI test, blood sampling, and ELISA. (*B*) Representative heatmaps of the CON, SUS, and RES mouse behavior during the SI test. (*C*) Social behavior in the CSDS model assessed by the SI test (CON: n = 16, SUS: n = 27, RES: n = 18, F (2, 58) = 91.70, *P* < 0.0001). (*D*) Distance moved during the SI test (no target) (CON: n = 16, SUS: n = 27, RES: n = 18, F (2, 58) = 7.518, *P* = 0.0012). (*E*) Total IgG antibody concentrations in sera (CON: n = 16, SUS: n = 27, RES: n = 18, F (2, 58) = 6.454, *P* = 0.0029). (*F*) Correlation between SI ratio and serum IgG antibody concentrations (CON: n = 16, SUS: n = 27, RES: n = 18, r = −0.2687, *P* = 0.0363). Data represented as mean ± SEM were analyzed by one-way ANOVA with Bonferroni post hoc test (*P < 0.05, **P < 0.01, ****P < 0.0001, ns: not significant). Correlation was evaluated by Pearson correlation analysis.

To explore adaptive immune responses in a mouse model of chronic psychosocial stress, C57BL/6J mice underwent 10 d of CSDS followed by social interaction (SI) testing (Fig. 1*A*). Stressed mice were classified as SUS or RES based on their SI ratio, calculated as the ratio of the time spent interacting with and without a social target mouse (Fig. 1 *B* and *C*). Consistent with our previous findings, both SUS and RES mice moved shorter distances during the SI test in the absence of a social target when compared with unstressed control (CON) mice; however, no significant differences were observed in locomotion between SUS and RES mice (Fig. 1*D*). We next analyzed total IgG antibody concentrations in sera via an enzyme-linked immunosorbent assay (ELISA). The sera from SUS mice showed significantly higher IgG concentrations than sera from CON mice (Fig. 1*E*). In addition, IgG concentrations negatively correlated with SI ratio (Fig. 1*F*). These results suggest that social stress induces antibody production, which may contribute to social avoidance behavior.

### Social Stress Induces Antibody Responses in Brain-draining Lymph Nodes.

**Fig. 2.**
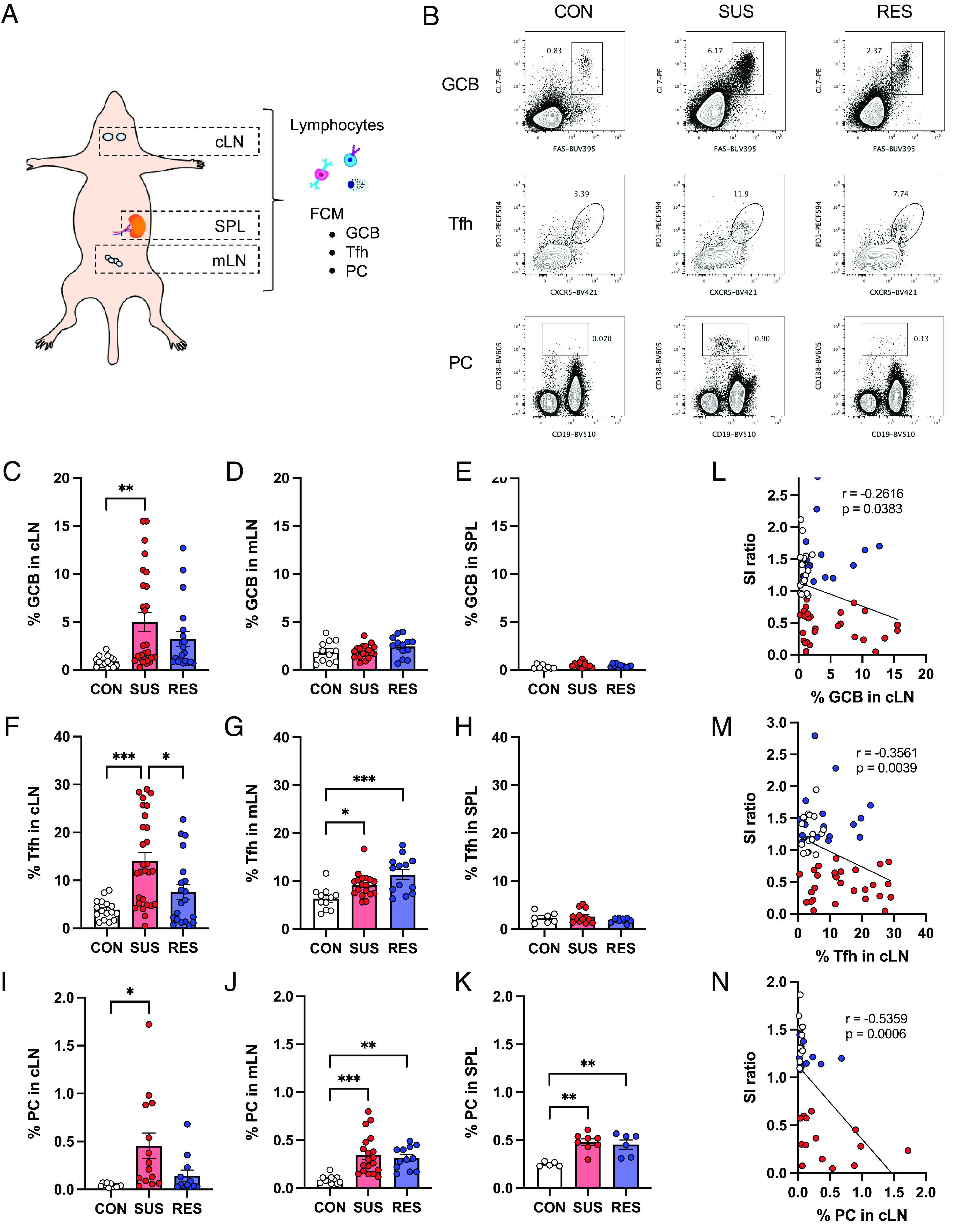
Preferential induction of immune cell populations controlling the germinal center reaction in brain-draining lymph nodes from SUS mice. (*A*) Anatomical diagram showing the locations of lymphoid organs assessed by FCM. (*B*) Representative FCM plots of GCB, Tfh, and PC in cLN from CON, SUS, and RES mice. (*C–K*) Percentages of GCB in (*C*) cLN (CON: n = 15, SUS: n = 27, RES: n = 20, F (2, 59) = 5.44, *P* = 0.0068), (*D*) mLN (CON: n = 12, SUS: n = 18, RES: n = 13, F (2, 40) = 1.478, *P* = 0.2402), and (*E*) SPL (CON: n = 8, SUS: n = 12, RES: n = 11, F (2, 28) = 3.254, *P* = 0.0536). Percentages of Tfh in (*F*) cLN (CON: n = 16, SUS: n = 28, RES: n = 20, F (2, 61) = 10.69, *P* = 0.0001), (*G*) mLN (CON: n = 11, SUS: n = 18, RES: n = 13, F (2, 39) = 8.767, *P* = 0.0007), and (*H*) SPL (CON: n = 7, SUS: n = 13, RES: n = 10, F (2, 27) = 1.524, *P* = 0.2360). Percentages of PC in (*I*) cLN (CON: n = 11, SUS: n = 14, RES: n = 12, F (2, 34) = 5.562, *P* = 0.0081), (*J*) mLN (CON: n = 11, SUS: n = 19, RES: n = 12, F (2, 39) = 10.60, *P* = 0.0002), and (*K*) SPL (CON: n = 5, SUS: n = 8, RES: n = 6, F (2, 16) = 11.24, *P* = 0.0009). (*L–N*) Correlation between SI ratio and percentages of (*L*) GCB (CON: n = 16, SUS: n = 27, RES: n = 20, r = −0.2616, *P* = 0.0383), (*M*) Tfh (CON: n = 16, SUS: n = 28, RES: n = 20, r = −0.3561, *P* = 0.0039), and (*N*) PC (CON: n = 11, SUS: n = 14, RES: n = 12, r = −0.5359, *P* = 0.0006) in cLN. Data represented as mean ± SEM were analyzed by one-way ANOVA with Bonferroni post hoc test (*P < 0.05, **P < 0.01, ***P < 0.001). Correlations were evaluated by Pearson correlation analysis. The *P* value threshold adjusted to *P* < 0.016 (*L*, *M*, and *N*).

To investigate whether antibody responses were induced in specific lymphoid organs after CSDS, we analyzed immune cell populations involved in the germinal center reaction. cLN, mLN, and SPL were collected 48 h after the last defeat, and analyzed for GCB, Tfh, and PC populations via flow cytometry (FCM) (Fig. 2 *A* and *B* and *SI Appendix*, Fig. S1). We observed a significant increase in the percentage of GCB only in the cLN from SUS mice (Fig. 2 *C*–*E*). Following CSDS, the percentages of Tfh significantly increased in the cLN and mLN but not in the SPL. Notably, cLN were the only lymphoid organs which showed a significant increase in Tfh only in SUS mice compared with that of CON mice (Fig. 2 *F*–*H*). In particular, the percentage of Tfh in the cLN from SUS mice was significantly higher than in both RES and CON mice (Fig. 2*F*). Meanwhile, the percentage of PC increased in all lymphoid organs, but was particularly pronounced in the cLN of SUS mice, showing a 17-fold increase compared with that in the cLN from CON mice (Fig. 2 *I*–*K*). In addition, correlations between immune cell population frequencies and depression-like behavior were most pronounced in the cLN (Fig. 2 *L*–*N* and *SI Appendix*, Fig. S2 *A*–*F*). Together, these results highlight that social stress strongly induces antibody responses in brain-draining lymph nodes specifically in SUS mice.

### Brain-reactive Antibodies Are Induced in Sera from SUS Mice and Humans with High Anhedonia.

**Fig. 3.**
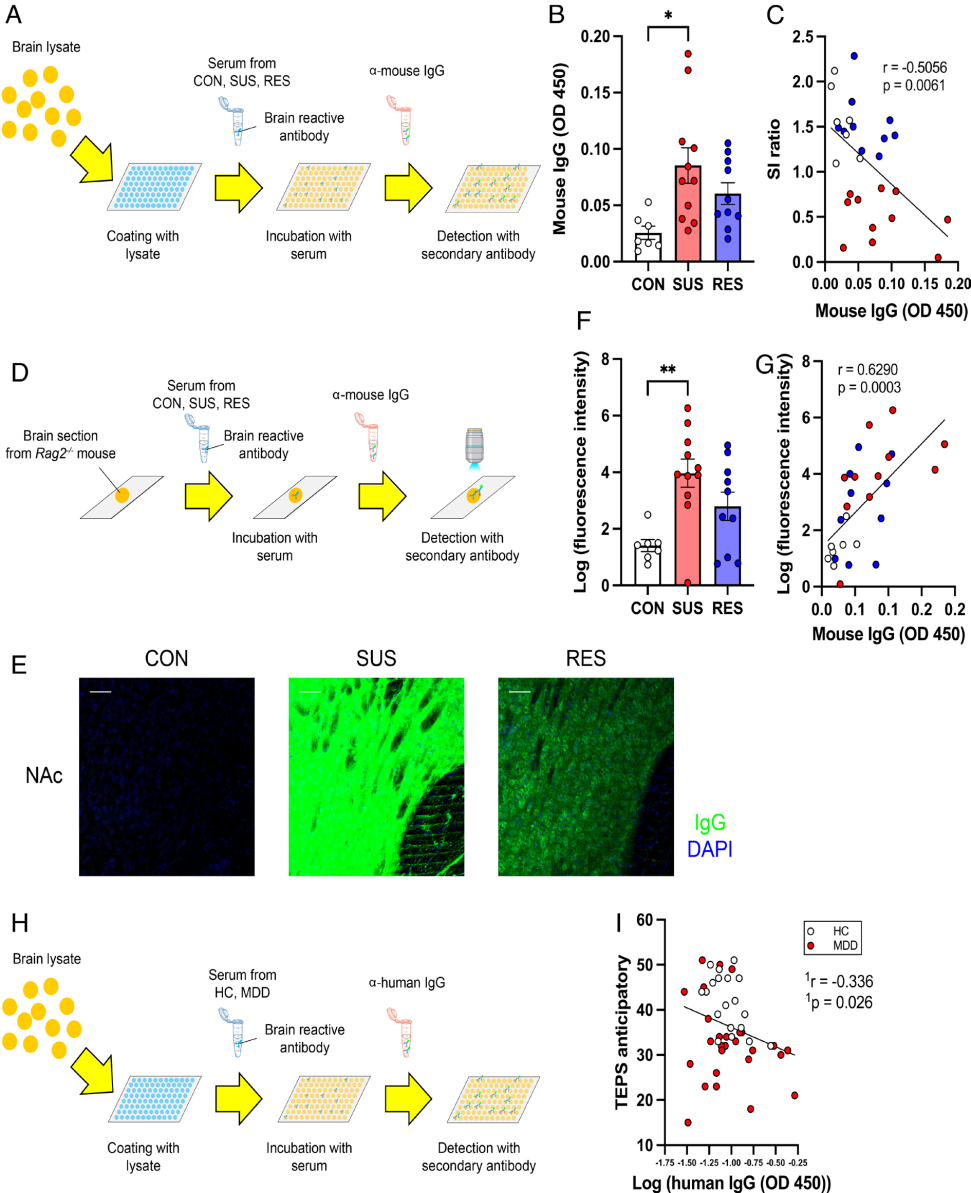
Increase in brain-reactive antibodies in sera from SUS mice and humans with high anhedonia. (*A*) Experimental outline: Schematic of detection of brain lysate-reactive IgG antibodies in sera from CON, SUS, and RES mice by ELISA. (*B*) Quantification of brain lysate-reactive IgG antibodies in sera (CON: n = 7, SUS: n = 11, RES: n = 10, F (2, 25) = 5.123, *P* = 0.0137). (*C*) Correlation between brain lysate-reactive IgG antibodies in sera and SI ratio (CON: n = 7, SUS: n = 11, RES: n = 10, r = −0.5056, *P* = 0.0061). (*D*) Experimental outline: Schematic of detection of brain section-reactive IgG antibodies in sera by indirect immunofluorescence. (*E*) Staining of brain sections around the NAc from immune-deficient *Rag2^−/−^* mice with sera from CON, SUS, and RES mice (green: IgG, blue: DAPI, Scale bar: 50 μm). (*F*) Quantification of fluorescence intensity (CON: n = 7, SUS: n = 11, RES: n = 10, F (2, 25) = 6.849, *P* = 0.0042). (*G*) Correlation between fluorescence intensity of brain sections stained with sera and levels of brain lysate-reactive serum IgG antibodies (CON: n = 7, SUS: n = 11, RES: n = 10, r = 0.6290, *P* = 0.0003). Data represented as mean ± SEM were analyzed by one-way ANOVA with Bonferroni post hoc test (*P < 0.05, **P < 0.01). Correlations were evaluated by Pearson correlation analysis. The *P* value threshold adjusted to *P* < 0.025 (*C* and *G*). (*H*) Experimental outline: Schematic of detection of brain lysate-reactive IgG antibodies in sera from HC and patients with MDD by ELISA. (*I*) Correlation between levels of brain lysate-reactive IgG antibodies in sera and the TEPS anticipatory (HC: n = 19, MDD: n = 28, ^1^r = −0.336, ^1^*P* = 0.026). The *P* value threshold adjusted to *P* < 0.0125 (*I*). ^1^The partial correlation was calculated to control for the potential confounding variables of age, gender, and body mass index (BMI).

Considering the preferential expansion of the T and B cell populations in brain-draining lymph nodes, we hypothesized that social stress induces antibody responses against antigens expressed in the brain, consequently promoting stress susceptibility. To test this, we quantified brain-reactive antibodies in the sera from mice following CSDS by ELISA (Fig. 3*A* and *SI Appendix*, Fig. S3 *A* and *B*). Sera from SUS mice showed significantly higher reactivity against brain lysates than those from CON mice (Fig. 3*B*). Furthermore, the levels of brain-reactive antibodies in the sera correlated with both social avoidance (Fig. 3*C*) and the percentage of immune cell populations controlling antibody production in the cLN (*SI Appendix*, Fig. S3 *C* and *D*). We next stained brain sections from immune-deficient *Rag2^−/−^* mice, which lack endogenous antibodies, with sera from CSDS-exposed mice and detected bound antibodies using anti-mouse IgG secondary antibodies (Fig. 3*D*). The NAc, PFC, and HIP were selected for analysis since previous findings indicated that stress can alter blood-brain barrier permeability and increase peripheral immune cell infiltration in these regions. This led us to hypothesize that autoimmune responses in the periphery might be induced by stress and interact with the neurovascular unit within these regions. Among the samples collected, sera from SUS mice showed the strongest reactivity against brain sections from the NAc (Fig. 3*E*), PFC, and HIP (*SI Appendix*, Fig. S3*E*). Brain sections of the NAc stained with sera from SUS mice showed a significantly higher fluorescence intensity than those stained with sera from CON mice (Fig. 3*F*). Furthermore, the fluorescence intensity of the brain sections correlated with the percentage of GCB and Tfh in the cLN (*SI Appendix*, Fig. S3 *F* and *G*). We also confirmed a correlation of brain reactivity of the sera detected by ELISA and immunofluorescence in brain sections incubated with sera (Fig. 3*G*). In addition, we measured the reactivity of sera against proteins expressed in the brain via western blot (*SI Appendix*, Fig. S3*H*). Specific bands were detected when membranes bound to proteins prepared from NAc, PFC, and HIP lysates were incubated with serum from SUS mice. The band patterns differed between lanes with proteins from different brain regions, indicating that there may be multiple targets of autoantibodies induced by stress (*SI Appendix*, Fig. S3*I*). To investigate the clinical relevance of these findings, we performed an exploratory study in humans with MDD (Table 1). We analyzed IgG concentrations and levels of brain-reactive antibodies in clinical samples of sera from healthy controls (HC) and patients with MDD. Using the Temporal Experience of Pleasure Scale (TEPS), a self-reported measure of pleasure experience or anhedonia (39), we evaluated correlations between brain-reactive antibodies in sera and TEPS anticipatory or consummatory pleasure (Fig. 3*H*). There was no significant difference in serum IgG concentrations between HC and MDD patients (*SI Appendix*, Fig. S4*A*). After correcting for multiple comparisons, levels of brain-reactive IgG antibodies showed a trend for correlation with TEPS anticipatory pleasure (Fig. 3*I*) and TEPS consummatory pleasure (*SI Appendix*, Fig. S4*B*). We also evaluated correlations with other clinical outcomes such as the Childhood Trauma Questionnaire (CTQ) and the Quick Inventory of Depressive Symptomatology (QIDS) (Table 1). While the results from this exploratory study did not reach statistical significance, we observed an encouraging trend (*SI Appendix*, Fig. S4 *C* and *D*). These results may provide important evidence indicating clinical relevance of autoimmune responses against the brain associated with stress and anhedonia.

**Table 1.** Sociodemographic variables and clinical data

	HC (n = 19)	MDD (n = 28)	Statistics
Age	39.95 ± 9.65	33.43 ± 9.71	t(45) = 2.264, *P* = 0.0284
Gender (m/f)	(10/9)	(11/17)	χ2 = 0.816, *P* = 0.390
BMI	25.72 ± 3.79	24.57 ± 5.48	t(45) = 0.7892, *P* = 0.4341
TEPS anticipatory	41.58 ± 6.27	32.82 ± 8.99	t(45) = 3.676, *P* = 0.0006
TEPS consummatory	35.95 ± 6.08	31.70 ± 9.03	t(44) = 1.781, *P* = 0.0819
CTQ total score	32.42 ± 9.82	46.70 ± 14.75	t(44) = 3.679, *P* = 0.0006
QIDS	1.63 ± 1.71	13.54 ± 4.80	t(45) = 10.35, *P* < 0.0001

Statistics: two-tailed Student’s *t* test (for age, BMI, TEPS anticipatory, TEPS consummatory, CTQ total score, and QIDS), Pearson’s chi-squared test (Gender). Abbreviations: BMI: body mass index; f: female; HC: healthy controls; m: male; MDD: major depressive disorder; TEPS: Temporal Experience of Pleasure Scale; CTQ: Childhood Trauma Questionnaire; QIDS: Quick Inventory of Depressive Symptomatology.

### IgG Antibodies Accumulate in the Brain Vasculature after CSDS.

**Fig. 4.**
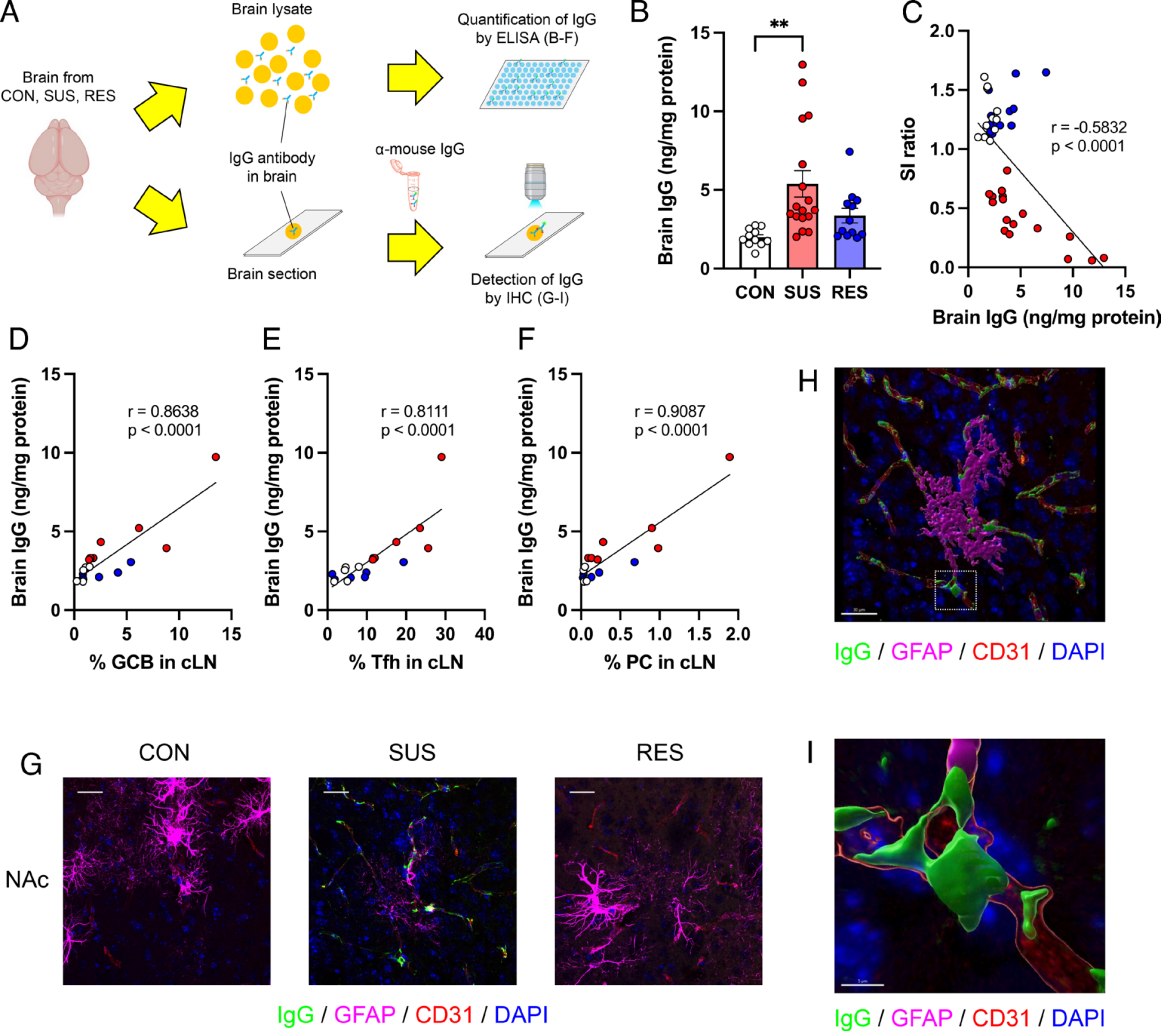
Accumulation of IgG antibodies in the brain of SUS mice. (*A*) Experimental outline: Schematic of the analysis of IgG antibodies in the brain by ELISA and IHC. (*B*) Quantification of IgG antibody concentrations in brain lysates from CON, SUS, and RES mice (CON: n = 11, SUS: n = 17, RES: n = 12, F (2, 37) = 6.686, *P* = 0.0033). (*C*) Correlation between SI ratio and brain IgG antibody concentrations (CON: n = 11, SUS: n = 17, RES: n = 12, r = −0.5832, *P* < 0.0001). (*D–F*) Correlations between brain IgG antibody concentrations and percentages of (*D*) GCB (CON: n = 5, SUS: n = 7, RES: n = 6, r = 0.8638, *P* < 0.0001), (*E*) Tfh (CON: n = 5, SUS: n = 7, RES: n = 6, r = 0.8111, *P* < 0.0001), and (*F*) PC (CON: n = 5, SUS: n = 7, RES: n = 6, r = 0.9087, *P* < 0.0001) in cLN. (*G*) Detection of IgG antibodies in brain sections around NAc from CON, SUS, and RES mice (green: IgG, magenta: GFAP, red: CD31, blue: DAPI, Scale bar: 25 μm). (*H* and *I*) 3D reconstructed images of a brain section from a SUS mouse showing IgG staining colocalized with brain vascular cell markers [green: IgG, magenta: GFAP, red: CD31, blue: DAPI, Scale bar: (*H*) 30 μm, (*I*) 5 μm]. The area inside the white frame in *H* is shown in *I*. Data represented as mean ± SEM were analyzed by one-way ANOVA with Bonferroni post hoc test (**P < 0.01). Correlations were evaluated by Pearson correlation analysis. The *P* value threshold adjusted to *P* < 0.016 (*D*, *E*, and *F*).

To investigate the presence of IgG antibodies in the brain, we prepared brain lysates and brain sections from PBS-perfused mice (to remove potential IgG antibodies from circulation) after CSDS and analyzed IgG antibodies quantitatively by ELISA and qualitatively using immunohistochemistry (IHC) (Fig. 4*A* and *SI Appendix*, Fig. S5 *A* and *B*). Brain lysates from SUS mice showed significantly higher IgG antibody concentrations than those from CON mice (Fig. 4*B*). Furthermore, the IgG antibody concentrations in the brain negatively correlated with SI behavior (Fig. 4*C*). We also performed correlation analyses between social avoidance behavior and immune parameters for each group. Importantly, IgG antibody levels in brain tissue correlated very strongly with social avoidance behavior in SUS mice (*SI Appendix*, Fig. S6*A*), whereas, peripheral immune parameters did not (*SI Appendix*, Fig. S6 *B*–*F*). Interestingly, the levels of IgG antibodies in the brain correlated with the percentages of immune cell populations controlling antibody production in the cLN (Fig. 4 *D*–*F*). Next, brain sections containing the NAc (Fig. 4*G*), PFC, and HIP (*SI Appendix*, Fig. S5*C*) from each group were stained with anti-mouse IgG secondary antibodies to detect antibodies in the brain. To visualize the cellular localization of IgG antibodies, we costained with markers of neurons, vascular cells (endothelial cells, pericytes, and vascular smooth muscle cells), and glial cells (astrocytes, microglia, and oligodendrocytes) (40). 3D reconstructed images from the NAc showed IgG antibodies were primarily detected in areas costained with cells of the vascular system, such as CD31-positive endothelial cells, and GFAP-positive astrocytes associated with the neurovascular unit in brain sections from SUS mice (Fig. 4 *H* and *I*). These data suggest that CSDS induces brain-reactive antibodies against components of the neurovascular unit and may contribute to stress susceptibility.

### B Cell Depletion before CSDS Promotes Stress Resilience.

**Fig. 5.**
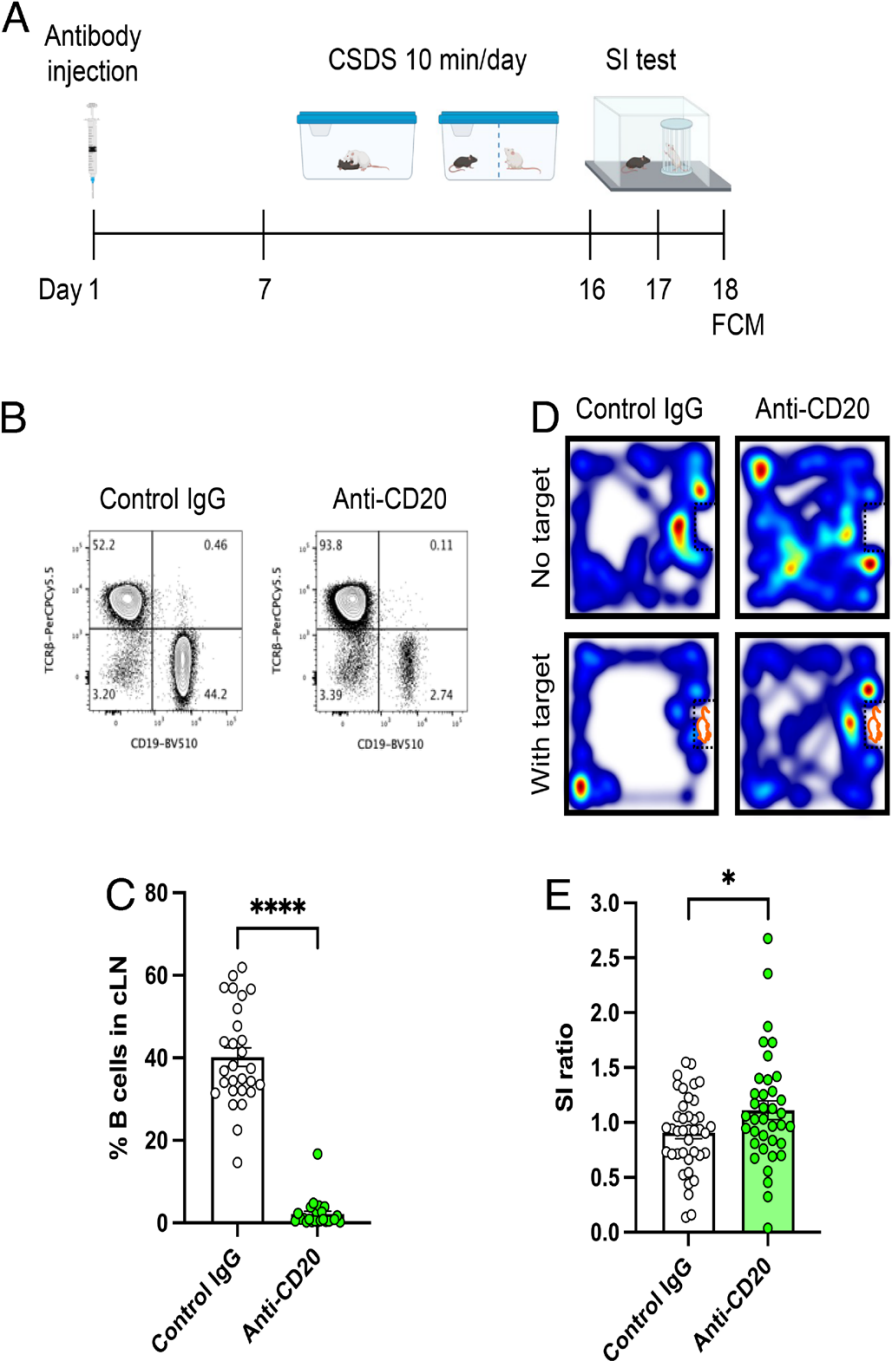
Contribution of B cells to depression-like behavior in the CSDS model. (*A*) Experimental outline: B cell depletion followed by CSDS, SI test and FCM. (*B*) Representative FCM plots of lymphocytes from cLN at day 18. (*C*) Analysis of B cell depletion efficiency (Control IgG: n = 28, Anti-CD20: n = 24, t (50) = 15.04, *P* < 0.0001). (*D*) Representative heatmaps of mouse behavior during the SI test. (*E*) Effects of B cell depletion on SI ratio in the CSDS model (Control IgG: n = 40, Anti-CD20: n = 37, t (75) = 2.066, *P* = 0.0423). Data represented as mean ± SEM were analyzed by unpaired *t* test (*P < 0.05, ****P < 0.0001).

We further investigated the contribution of antibody responses to stress-susceptibility by depleting antibody-producing B cells. Production of autoantibodies is mediated by clonal expansion of autoreactive B cells in the germinal center (41). Thus, we depleted B cells by administering an anti-CD20 antibody before exposing mice to CSDS and then tested social interaction behavior (Fig. 5*A*). We confirmed the absence of B cells in secondary lymphoid organs using FCM 7 d after anti-CD20 antibody injection in a separate cohort of mice (*SI Appendix*, Fig. S7*A*). Importantly, we observed a marked reduction in the population of B cells in the cLN by anti-CD20 treatment 48 h after the last defeat, indicating that the absence of B cells persisted throughout the experiment (Fig. 5 *B* and *C*). Following CSDS, the B cell-depleted group showed a significantly higher SI ratio than the control IgG-treated group (Fig. 5 *D* and *E* and *SI Appendix*, Fig. S7*B*), indicating that B cell depletion promoted stress resilience. These data suggest that B cells contribute to stress susceptibility in the CSDS model.

## Discussion

In this study, we demonstrated a role for the adaptive immune system in stress susceptibility and depression, possibly through the production of autoantibodies targeting antigens in the brain. Previous studies have revealed the critical roles of the innate immune system, particularly monocyte/macrophage-dependent mechanisms in depression. Complementing these findings, our study highlights the important contributions of adaptive immune abnormalities. We observed that social stress strongly induces germinal center responses in brain-draining lymph nodes and production of brain-reactive antibodies that bind to cells of the neurovascular unit in brain regions involved in stress and depression, and these effects correlate with behavioral abnormalities. We also show that B cell depletion promotes behavioral resilience against CSDS. These data suggest that CSDS induces autoimmune responses against the brain, which contribute to the pathogenesis of depression-like behavior (*SI Appendix*, Fig. S8). Results obtained from this study may provide important evidence for future clinical studies to test causal hypotheses regarding stress-induced autoimmune responses against the brain in a host of stress-related disorders.

Overactivation of immune cell populations such as Tfh, GCB, and PC have been linked to autoimmune diseases (26). cLN have been identified as sites of brain antigen delivery from the cerebrospinal fluid through lymphatic vessels in the meninges (42). Recent reports have indicated involvement of cLN in animal models of CNS diseases such as multiple sclerosis, Alzheimer’s disease, and stroke (43–45). This study shows that social stress induces the activation of antibody responses in the cLN, including strong induction of GCB, Tfh, and PC in cLN from SUS mice. Our results suggest that stress-induced activation of immune cells in these lymph nodes may contribute to depression-like behaviors. Consistent with a recent study showing cerebrovascular dysfunction in the CSDS model (46), results of the present study reveal that IgG antibodies accumulate around the blood vessels in brain sections from SUS mice. This finding suggests that proteins expressed in the brain vasculature are candidate targets of autoantibodies induced by social stress, although the exact target antigens of these autoantibodies remain unknown. Identification of these targets will help elucidate the mechanisms by which stress-induced autoantibodies mediate depression. Technologies, such as cloning of monoclonal antibodies, can be used to identify brain targets of autoantibodies (47). Given that we detected dramatic increases of PC in brain draining lymph nodes in stress-susceptible mice, single-cell analysis of these PC might lead to identification of monoclonal antibodies targeting brain antigens. In addition, the clinical relevance of autoimmune targets could be investigated to confirm reactivity in serum and CSF from depressed subjects.

We have reported preexisting individual differences of inflammatory status that predict stress susceptibility (19). Association of prestress antibody levels with behavioral abnormalities after stressing mice is an important question that will need to be evaluated in future studies. Further, although B cell depletion induced stress resilience, the effect was relatively mild. There are both effector and regulatory B cell subsets (48) and elimination of all B cells might adversely affect the social behavior since B cells with regulatory function are required for controlling inflammatory responses. As discussed, we believe identification of targets of autoantibodies induced by stress and investigation of the target antigen-mediated mechanisms will greatly help understand how antibody responses contribute to the behavior.

Adding translational value to our studies, we found an association between peripheral levels of brain-reactive antibodies and severity of anhedonia in humans. Recent reports have indicated dysfunction of adaptive immune responses, including autoimmunity, in adolescents and young adults with suicidal behavior (49) and activation of B cells in postpartum depression (50); these reports reinforce the clinical relevance of our study findings. One limitation of the current human study is its exploratory nature and small sample size. Future studies that are adequately powered by taking into account the heterogeneity of MDD—i.e., only about 25 to 30% of patients with MDD display chronic low-grade inflammation—should be performed to further interrogate how autoimmune responses relate to different clinical features, particularly anhedonia.

Overall, our results suggest that patients with MDD might benefit from the identification of disease-relevant autoantibodies, eventually leading to the development of therapeutic strategies targeting autoimmunity, particularly for alleviating symptoms of anhedonia.

## Materials and Methods

### Mice.

Six- to seven week-old male C57BL/6J (Stock#: 000664) mice were purchased from The Jackson Laboratory. Retired breeder male CD-1 mice over 4 mo old were purchased from Charles River Laboratories. *Rag2^−/−^* mice were kindly provided by Miriam Merad. All mice were maintained with ad libitum access to food and water. All experiments were performed in accordance with the National Institutes of Health Guide for the Care and use of Laboratory animals and approved by the Icahn School of Medicine at Mount Sinai Institutional Animal Care and Use Committee.

### CSDS.

CSDS was performed as previously described (18). CD-1 mice with aggressive behavior (CD-1 AGG) were selected by intermale social interactions over three consecutive days based on previously described criteria and housed in a social defeat cage (26.7 cm width × 48.3 cm depth × 15.2 cm height, Allentown Inc.) 24 h before the start of defeats on one side of a clear perforated Plexiglas divider (0.6 cm × 45.7 cm × 15.2 cm, Nationwide Plastics). Eight-week-old experimental C57BL/6J mice were subjected to physical interactions with an unfamiliar CD-1 AGG mouse for 10 min once per day for 10 consecutive days. After antagonistic interactions with the CD-1 AGG mice, experimental mice were removed and housed on the opposite side of the divider, allowing sensory contact over the subsequent 24-h period. Unstressed control (CON) mice were housed two per cage on either side of a perforated divider without being exposed to the CD-1 AGG mice. Experimental mice were singly housed after the last bout of defeat and the SI test was conducted 24 h later.

### SI Test.

SI testing was performed as previously described (18). First, mice were placed in a Plexiglas open-field arena with a small wire animal cage placed at one end. Movements were monitored and recorded automatically for 150 s with an automated tracking system (Ethovision 11.0 Noldus Information Technology) to determine baseline exploratory behavior and locomotion in the absence of a social target (CD-1 AGG mouse). At the end of 150 s, the mouse was removed, and the arena was cleaned with 70% ethanol. Next, exploratory behavior in the presence of a novel social target inside the small wire animal cage was measured for 150 s, and time spent in the interaction zone was analyzed. SI ratio was calculated by dividing the time spent in the interaction zone when the AGG was present by the time spent in the interaction zone when the AGG was absent. All mice with a SI ratio < 1.0 were classified as SUS and all mice with a SI ratio ≥ 1.0 were classified as RES.

### FCM.

Single-cell suspensions were prepared from lymphoid organs (cLN, mLN, and spleen) in a staining buffer (0.5% BSA, 2 mM EDTA in PBS). Red blood cells were removed from splenocytes by treatment with 1× lysing buffer (BD Pharm Lyse). After blocking of FcγR (1:200 dilution, 4 °C, 30 min), cell surface markers were stained with fluorochrome-labeled antibodies (1:200–400 dilution, 4 °C, 30 min). For staining of intracellular markers, cells were fixed and permeabilized using True-Nuclear Transcription Factor Buffer Set (Biolegend) following manufacturers’ instructions. Intracellular markers were stained with fluorochrome-labeled antibodies (1:200–400 dilution, 4 °C, 30 min). Dead cells were stained with Fixable Viability Dye eFluor®780 (eBioscience) and excluded from the analyses. Antibodies and other reagents used for the analyses are summarized in *SI Appendix*, Tables S1 and S2. Stained cells were acquired on a BD LSRII flow cytometer and obtained data were analyzed with FlowJo software version 10.6.2 (Tree Star).

### B Cell Depletion.

Control IgG antibody (250 μg, clone: RTK4530, Biolegend) or anti-CD20 antibody (250 μg, clone: SA271G2, Biolegend) were retroorbitally injected into seven-week-old C57BL/6J mice 1 wk before starting CSDS. Efficiency of B cell depletion was evaluated by analyzing CD19-positive B cells in lymphoid organs by FCM.

### IHC.

Brains were collected after transcardial perfusion with ice-cold PBS followed by 4% paraformaldehyde (PFA, Electron Microscopy Sciences). Collected brains were postfixed with 4% PFA (4 °C, overnight) and NAc, PFC, and HIP brain sections were prepared at 50 μm thickness by vibratome (Leica). After blocking with 3% normal donkey serum with 0.3% Triton X-100 (Sigma) in PBS (RT, 2 h), sections were incubated with primary antibodies (anti-GFAP antibody; 1:1 dilution, anti-CD31 antibody; 1:400 dilution, 4 °C, overnight) followed by staining with secondary antibodies (1:300 dilution, RT, 1 h). Nuclei were stained by DAPI (0.5 μg/mL, Molecular Probes). 3D rendered images were constructed by using IMARIS 9.9 software to create surfaces of each stain based on a threshold applied to all images. Antibodies and other reagents used for the analyses are summarized in *SI Appendix*, Tables S1 and S2. The brain sections were analyzed with a Zeiss LSM 780 confocal microscope.

### Visualization and Detection of Brain-Reactive Antibodies.

To detect brain-reactive IgG in sera, indirect immunofluorescence analyses were performed. Brain sections (50 μm) from NAc, PFC, and HIP were prepared from *Rag2^−/−^* mice. After blocking as described above, sections were incubated with sera from CON, SUS, and RES mice (1:50 dilution, 4 °C, overnight), and bound antibodies were detected and captured with anti-mouse IgG secondary antibodies (1:300 dilution, RT, 1 h) (Jackson ImmunoResearch). The brain sections were imaged and analyzed with a Zeiss LSM 780 confocal microscope. Fluorescence intensity from images were quantified using ImageJ. Briefly, images were first converted to 8-bit grayscale and the region of interest was selected using the drawing/selection tools to create a rectangle, then set as Macro. Macro was then randomly placed to measure fluorescence intensity by calculating the mean value of three to five representative fields per sample.

### Brain Lysate Preparation.

Brains were collected after transcardial perfusion with ice-cold PBS. Half-brains from mice were mashed in 300 μL of corresponding buffers using the plunger end of a 1-mL syringe. 1× TBS (Fisher) was used as a buffer to prepare lysates for detection of IgG antibodies in the brain lysates. PBS with protease inhibitors (cOmplete™, Mini, EDTA-free Protease Inhibitor Cocktail, Roche) was used as a buffer to prepare lysates for detecting brain lysate-reactive IgG antibodies in sera. Samples were further homogenized using a 1.5-mL pestle. Soluble fractions were collected after centrifugation. Total protein concentrations of the brain lysates were determined using a BCA Protein Assay Kit (Pierce).

### Western Blotting.

Blood samples were collected from mice and allowed to clot by leaving them undisturbed at room temperature. Sera were collected after centrifugation (2,000 × *g*, 4 °C, 10 min) and stored at −80 °C. Sera were analyzed for the presence of autoantibodies by western blotting as previously described (51) with some modifications. Briefly, brain lysates were separated by SDS-PAGE and transferred onto membranes (PVDF membrane, Bio-Rad Laboratories). After blocking (Blocking Buffer, ROCKLAND), membranes were incubated with sera (1:500 dilution, 4 °C, overnight), and bound antibodies were detected with a IRDye® 800CW-labeled anti-mouse IgG (H+L) secondary antibody (LI-COR) (1:5,000 dilution, RT, 40 min). The membranes were analyzed by a LI-COR Odyssey Infrared Imaging system (LI-COR Biosciences).

### Human Subjects.

Study participants with MDD and HC, as assessed by the Structured Clinical Interview for the Diagnostic and Statistical Manual of Mental Disorders–Fifth Edition (SCID-5) (52) were recruited through the Depression and Anxiety Center for Discovery and Treatment at the Icahn School of Medicine at Mount Sinai (ISMMS). The ISMMS review board approved the study, and written informed consent was obtained from all participants prior to any study procedure. Participants were compensated for their time and effort. Subjects provided demographic information and underwent a psychiatric evaluation using the SCID-5 conducted by trained study staff. Anhedonia was assessed using the TEPS (53), a well-validated self-report questionnaire, assessing both, consummatory and anticipatory anhedonia. A higher score on the TEPS indicates great experience of pleasure (less anhedonia). Total symptom severity was assessed by the QIDS scale (54) and a history of childhood trauma was assessed using the CTQ (55). All participants underwent biochemistry and hematological laboratory testing, urine toxicology, and pregnancy testing (if applicable). At the time of enrollment, all participants were free of medications known to affect the immune system for at least 2 wk. Participants were free of active infections or systemic illness. Subjects with concomitant unstable medical illnesses were excluded. Participants were free of current substances of abuse. On the day of blood draw, patients were fasted for at least 6 h. For serum isolation, blood was drawn into Vacutainer Gold Top 5 mL Silica Gel tubes (BD, #365968). Blood was allowed to clot for 30 min, then centrifuged at 1,300 × *g* for 15 min at 4 °C, then aliquoted and stored at −80 °C until further processing.

### ELISA.

Total IgG concentrations in sera and brain lysates were analyzed by ELISA following the manufacturers’ instructions. For quantification of brain lysate-reactive IgG antibodies in sera, brain lysates were diluted in an ELISA Coating Buffer (Biolegend) at 20 mg/mL and 50 mL was added to each well of a 96-well plate for coating (4 °C, overnight). Wells were blocked with 1% BSA and 0.1% Tween-20 in PBS (RT, 2 h). Mouse or human sera in 0.5% BSA and 0.05% Tween-20 in PBS (1:50 dilution) were added to the wells for incubation (RT, 2 h). Bound antibodies were detected with HRP-conjugated anti-mouse or human IgG detection antibodies (RT, 1h) followed by a color reaction with TMB substrate (RT, 15 min). The reaction was stopped by adding a stop solution and absorbance at 450 nm and 570 nm was measured. Values of absorbance at 570 nm were subtracted from those at 450 nm.

### Statistical Analyses.

Details of statistical analyses are described in the figure legends, including type of statistical analysis used, *P* values, and number of samples. Statistical analyses were performed using GraphPad Prism software (GraphPad Software Inc.) or SPSS (Version 24, IBM Corp., SPSS Inc., Chicago, IL). Samples that deviated from the mean by greater than 2-SD were identified as outliers and excluded from the analyses. For the analysis of the data involving human participants, normal distribution of the data was tested using the Kolmogorov–Smirnov Test. If data were not normally distributed, they were log transformed. Partial correlations were calculated to control for the potential confounding variables, age, gender and BMI. Level of statistical significance was set at *P* < 0.05. *P* value thresholds were adjusted for correlation analyses which required correction for multiple comparisons. Adjusted *P* value thresholds were specified in the figure legends.

## Supplementary Material

Appendix 01 (PDF)

Dataset S01 (XLSX)

## Data Availability

All study data are included in the article and/or supporting information.
