# Supplementary material for: Social stress induces autoimmune responses against the brain
Source: Proc Natl Acad Sci U S A. 2023 Nov 27;120(49):e2305778120. doi: 10.1073/pnas.2305778120 (PMC10710076; doi:10.1073/pnas.2305778120)
Supplement: Supplementary file 1 — Appendix 01 (PDF) [file pnas.2305778120.sapp.pdf]

PNAS

Supporting Information for
Social stress induces autoimmune responses against the brain

Yusuke Shimo, Flurin Cathomas, Hsiao-yun Lin, Kenny L. Chan, Lyonna F. Parise, Long Li, Carmen Ferrer-Pérez, Samer Muhareb, Sara Costi, James W. Murrough, and Scott J. Russo

Corresponding author: Scott J. Russo
Email: scott.russo@mssm.edu

This PDF file includes:

Figures S1 to S8
Tables S1 and S2

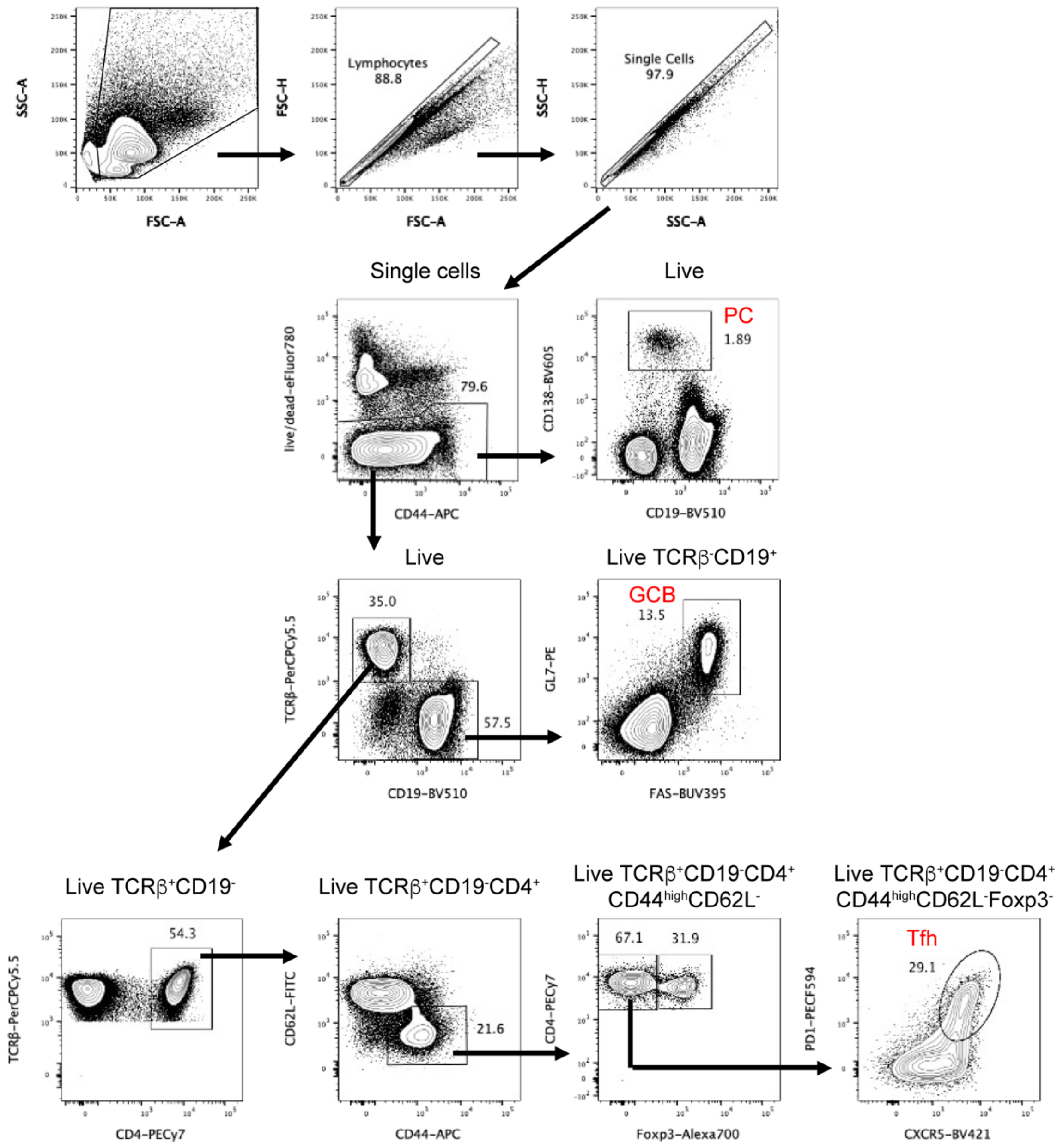

Fig. S1. Gating strategy of the flow cytometric analysis of germinal center B cells (GCB), follicular helper T cells (Tfh), and plasma cells (PC). Percentages of immune cell populations were analyzed as described: GCB: $GL7^{+}FAS^{+}$ in live $CD19^{+}TCR\beta^{-}$ cells; Tfh: $CXCR5^{high}PD1^{high}$ in live $TCR\beta^{+}CD19^{-}CD4^{+}CD44^{high}CD62L^{-}Foxp3^{-}$ cells; PC: $CD138^{+}$ in live cells.

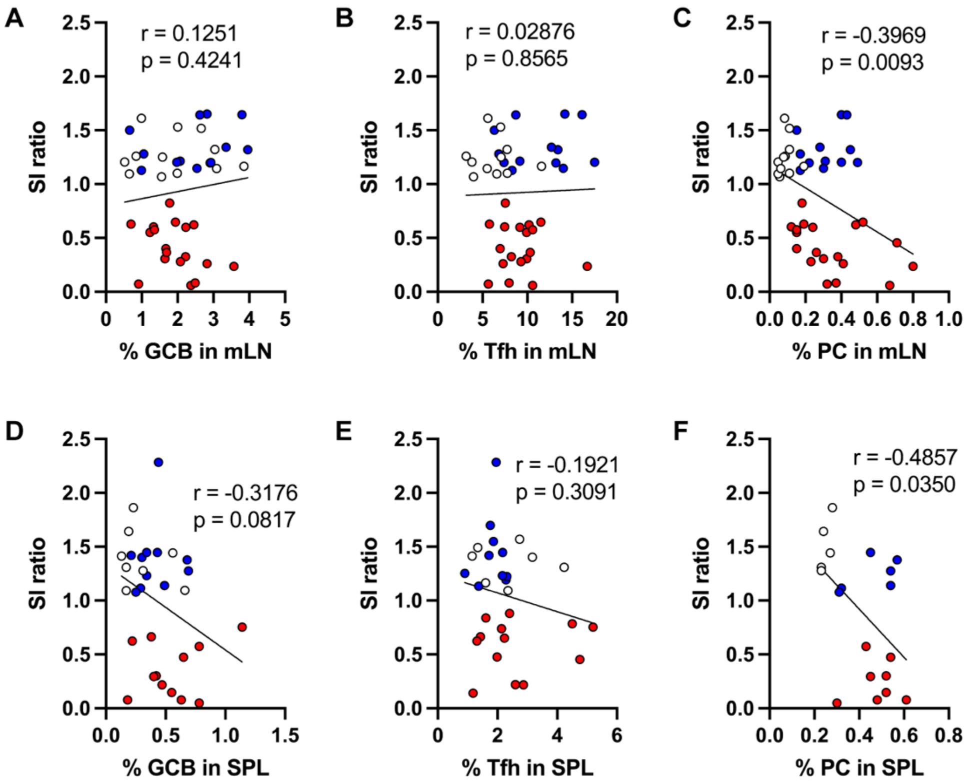

Fig. S2. Correlations between social behavior and immune cell populations in mesenteric lymph nodes (mLN) and spleen (SPL) from unstressed control (CON), stress-susceptible (SUS), and stress-resilient (RES) mice. Correlations between social interaction (SI) ratio and percentages of (*A*) germinal center B cells (GCB) (CON: n = 12, SUS: n = 18, RES: n = 13, r = 0.1251, *P* = 0.4241), (*B*) follicular helper T cells (Tfh) (CON: n = 11, SUS: n = 18, RES: n = 13, r = 0.02876, *P* = 0.8565), and (*C*) plasma cells (PC) (CON: n = 11, SUS: n = 19, RES: n = 12, r = -0.3969, *P* = 0.0093) in mLN. Correlations between SI ratio and percentages of (*D*) GCB (CON: n = 8, SUS: n = 12, RES: n = 11, r = -0.3176, *P* = 0.0817), (*E*) Tfh (CON: n = 7, SUS: n = 13, RES: n = 10, r = -0.1921, *P* = 0.3091), and (*F*) PC (CON: n = 5, SUS: n = 8, RES: n = 6, r = -0.4857, *P* = 0.0350) in SPL. Color: white = CON, red = SUS, blue = RES. Correlations were evaluated by Pearson correlation analysis. The *P* value threshold adjusted to $P < 0.016$ (*A*-*F*).

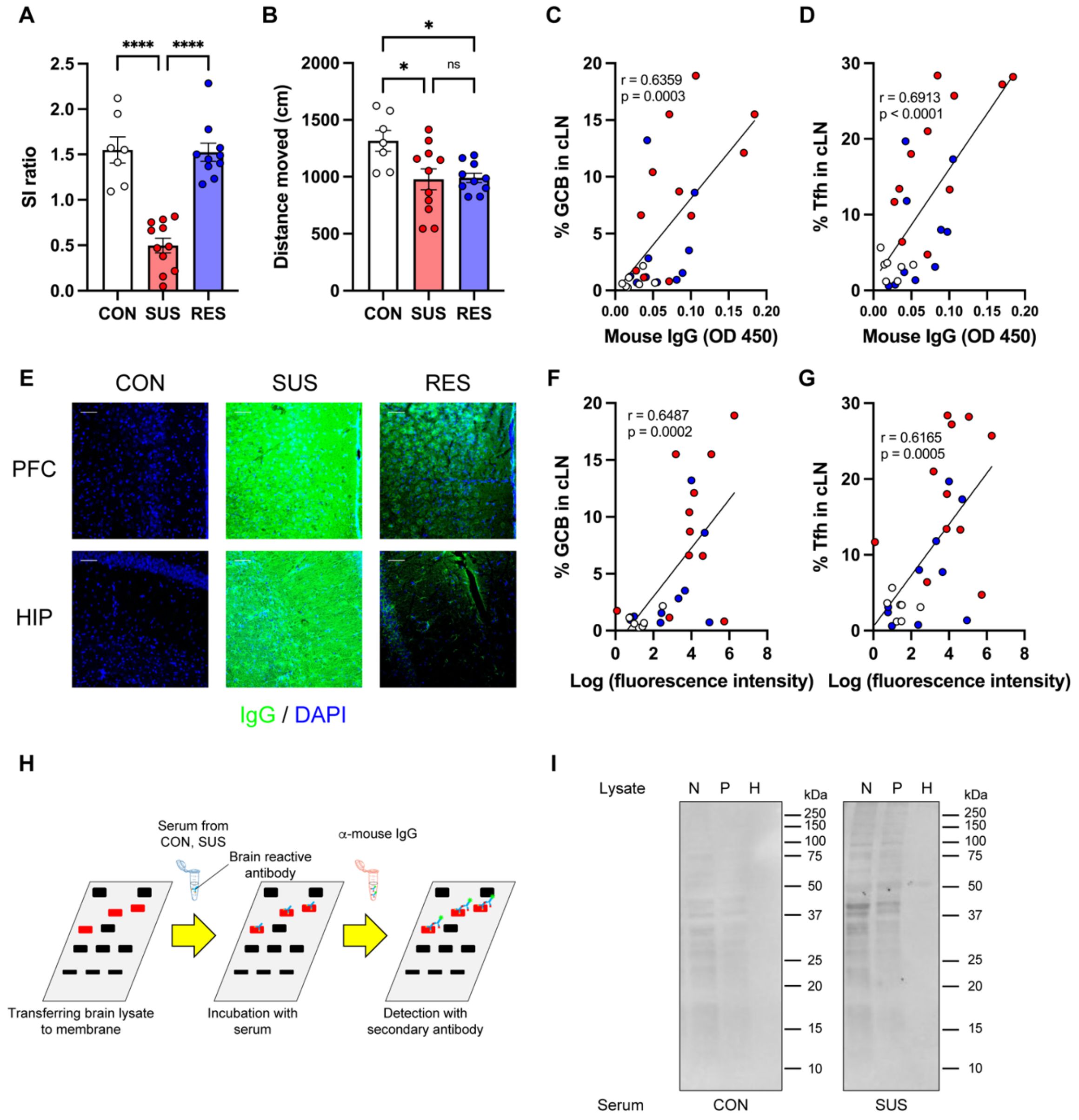

Fig. S3. Analysis of brain-reactive serum antibodies, and correlations with immune cell populations in cervical lymph nodes (cLN), and brain-reactive IgG antibodies in sera. (*A*) Social interaction (SI) ratio of unstressed control (CON), stress-susceptible (SUS), and stress-resilient (RES) mice (CON: n = 7, SUS: n = 11, RES: n = 10, F (2, 25) = 35.99, *P* < 0.0001). (*B*) Distance moved during the SI test (no target) (CON: n = 7, SUS: n = 11, RES: n = 10, F (2, 25) = 5.111, *P* = 0.0138). (*C* and *D*) Correlation between levels of brain lysate-reactive serum IgG antibodies and percentages of (*C*) germinal center B cells (GCB) (CON: n = 7, SUS: n = 11, RES: n = 10, r = 0.6359, *P* = 0.0003), (*D*) follicular helper T cells (Tfh) (CON: n = 7, SUS: n = 11, RES: n = 10, r = 0.6913, *P* < 0.0001). (*E*) Staining of brain sections around the prefrontal cortex (PFC) and hippocampus (HIP) from immune-deficient *Rag2*$^{-/-}$ mice with sera from CON, SUS, and RES mice (green: IgG, blue: DAPI, scale bar: 50 μm). (*F* and *G*) Correlation between fluorescence intensity of brain sections stained with sera and percentages of (*F*) GCB (CON: n = 7, SUS: n = 11, RES: n = 10, r = 0.6487, *P* = 0.0002), (*G*) Tfh (CON: n = 7, SUS: n = 11, RES: n = 10, r = 0.6165, *P* = 0.0005). (*H*) Schematic for detecting brain-reactive IgG antibodies in sera by Western blotting. (*I*) Specific bands detected on a membrane with brain lysates from the nucleus accumbens (N), prefrontal cortex (P), and hippocampus (H) incubated with serum from a SUS

mouse. Numbers in the figure indicate protein size (kDa). Data represented as mean ± standard error of the mean were analyzed by one-way ANOVA with Bonferroni post hoc test (*$P < 0.05$, ****$P < 0.0001$, ns: not significant). Correlations were evaluated by Pearson correlation analysis. The P value threshold adjusted to $P < 0.025$ (*C, D, F,* and *G*).

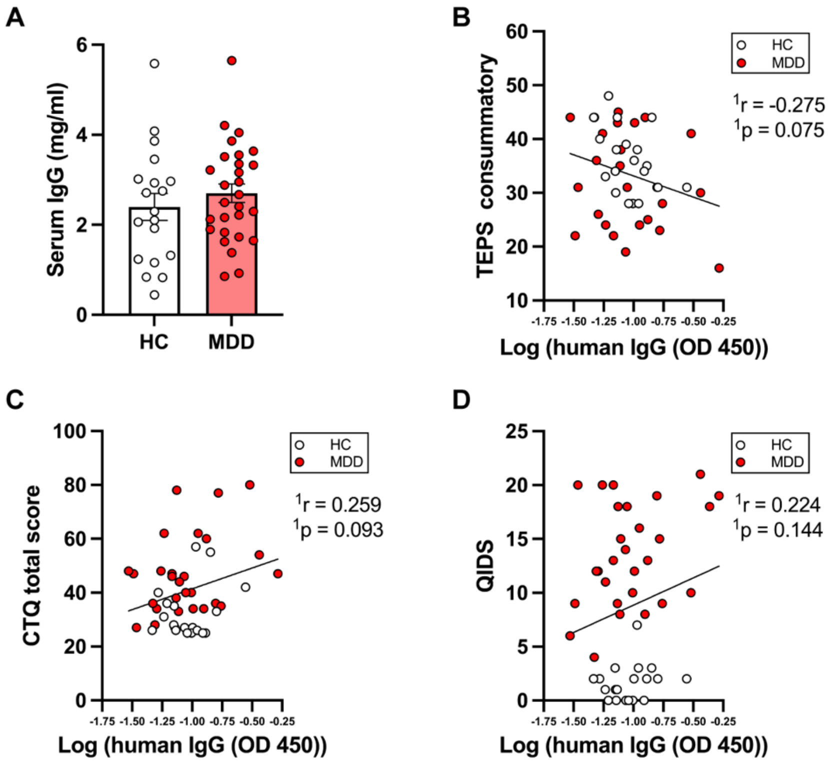

Fig. S4. Analyses of serum IgG concentrations and correlation between levels of brain-reactive serum IgG antibodies and clinical outcomes. (*A*) Total IgG antibody concentrations in sera from healthy controls (HC) and patients with major depressive disorder (MDD) (HC: n = 19, MDD: n = 28, t (45) = 0.8614, *P* = 0.3936). (*B*) Correlation between levels of brain lysate-reactive IgG antibodies in sera and the Temporal Experience of Pleasure Scale (TEPS) consummatory (HC: n = 19, MDD: n = 27, [1]r = -0.275, [1]*P* = 0.075). (*C*) Correlation between levels of brain lysate-reactive IgG antibodies in sera and the Childhood Trauma Questionnaire (CTQ) total score (HC: n = 19, MDD: n = 27, [1]r = 0.259, [1]*P* = 0.093). (*D*) Correlation between levels of brain lysate-reactive IgG antibodies in sera and the Quick Inventory of Depressive Symptomatology (QIDS) (HC: n = 19, MDD: n = 28, [1]r = 0.224, [1]*P* = 0.144). The *P* value threshold adjusted to $P < 0.0125$ (*B*, *C,* and *D*). Data represented as mean ± standard error of the mean were analyzed by unpaired t-test. [1]The partial correlation was calculated to control for the potential confounding variables of age, gender, and body mass index (BMI).

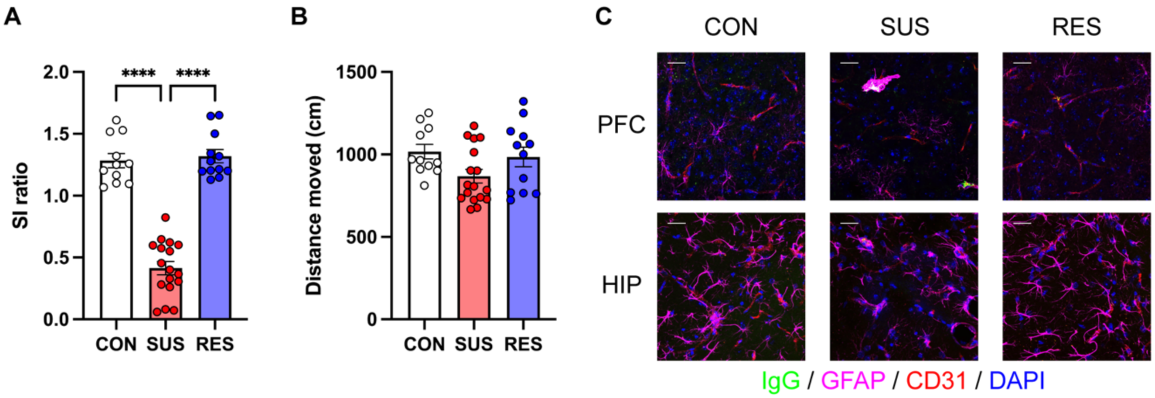

Fig. S5. Behaviors and representative brain section images for analyses of IgG antibodies in the brain from unstressed control (CON), stress-susceptible (SUS), and stress-resilient (RES) mice. (*A*) Social interaction (SI) ratio (CON: n = 11, SUS: n = 17, RES: n = 12, F (2, 37) = 93.46, $P < 0.0001$). (*B*) Distance moved during the SI test (no target) (CON: n = 11, SUS: n = 17, RES: n = 12, F (2, 37) = 2.876, $P = 0.0691$). (*C*) Staining of IgG antibodies in brain sections of prefrontal cortex (PFC), and hippocampus (HIP) from CON, SUS, and RES mice (green: IgG, magenta: GFAP, red: CD31, blue: DAPI, scale bar: 25 μm). Data represented as mean ± standard error of the mean were analyzed by one-way ANOVA with Bonferroni post hoc test (****$P < 0.0001$).

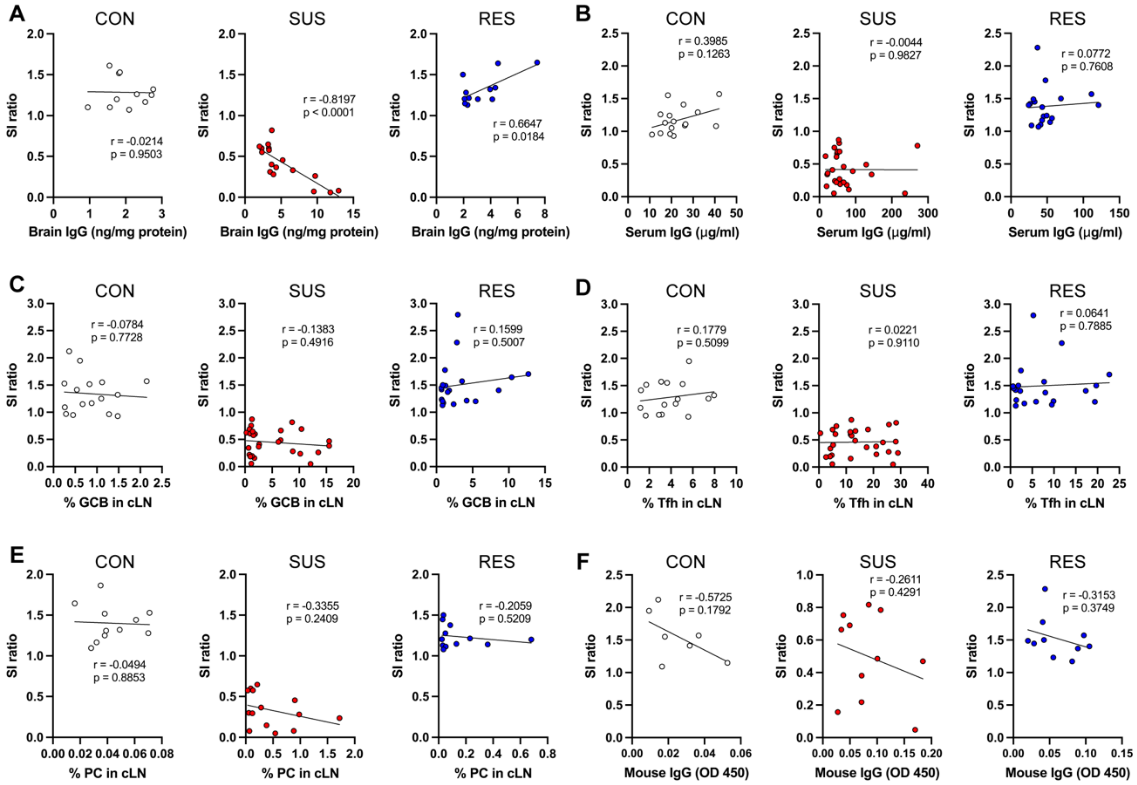

Fig. S6. Correlations between social behavior and immune parameters for each group. Correlations between social interaction (SI) ratio and (*A*) IgG concentrations in the brain (CON: n = 11, r = -0.0214, *P* = 0.9503, SUS: n = 17, r = -0.8197, *P* < 0.0001, RES: n = 12, r = 0.6647, *P* = 0.0184), (*B*) serum IgG concentrations (CON: n = 16, r = 0.3985, *P* = 0.1263, SUS: n = 27, r = -0.0044, *P* = 0.9827, RES: n = 18, r = 0.0772, *P* = 0.7608), (*C*) percentages of germinal center B cells (GCB) in cervical lymph nodes (cLN) (CON: n = 16, r = 0.0784, *P* = 0.7728, SUS: n = 27, r = -0.1383, *P* = 0.4916, RES: n = 20, r = 0.1599, *P* = 0.5007), (*D*) percentages of follicular helper T cells (Tfh) in cLN (CON: n = 16, r = 0.1779, *P* = 0.5099, SUS: n = 28, r = 0.0221, *P* = 0.9110, RES: n = 20, r = 0.0641, *P* = 0.7885), (*E*) percentages of plasma cells (PC) in cLN (CON: n = 11, r = -0.0494, *P* = 0.8853, SUS: n = 14, r = -0.3355, *P* = 0.2409, RES: n = 12, r = -0.2059, *P* = 0.5209), (*F*) brain-reactive serum IgG levels analyzed by enzyme-linked immunosorbent assay (CON: n = 7, r = -0.5725, *P* = 0.1792, SUS: n = 11, r = -0.2661, *P* = 0.4291, RES: n = 10, r = -0.3153, *P* = 0.3749). Correlations were evaluated by Pearson correlation analysis. The *P* value threshold adjusted to *P* < 0.016 (*C, D,* and *E*).

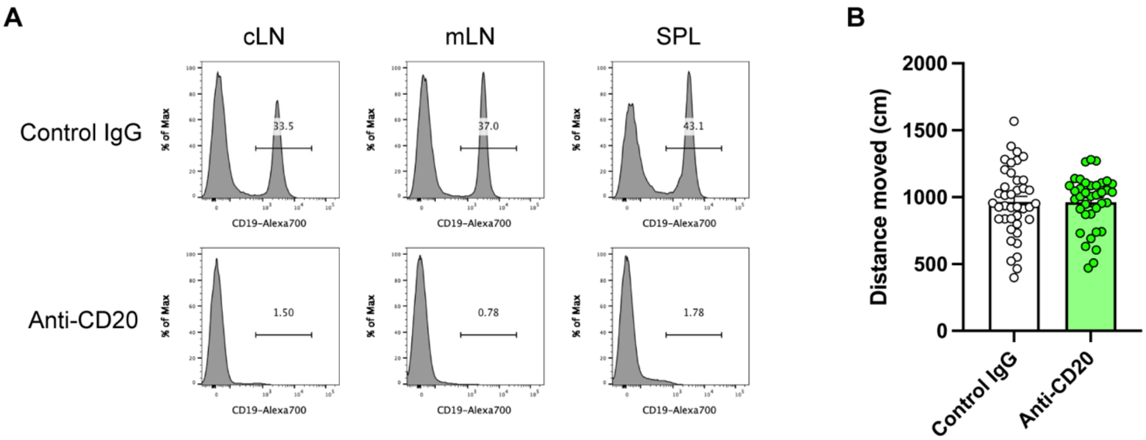

Fig. S7. Analyses of B cells in different lymphoid organs and behaviors in B cell depletion experiments. (*A*) Percentages of B cells in cervical lymph nodes (cLN), mesenteric lymph nodes (mLN), and spleen (SPL) 7 days after control IgG or anti-CD20 antibody treatments. (*B*) Distance moved during the social interaction (SI) test (no target) (Control IgG: n = 40, Anti-CD20: n = 37, t (75) = 0.03703, *P* = 0.9706). Data represented as mean ± standard error of the mean were analyzed by unpaired t-test.

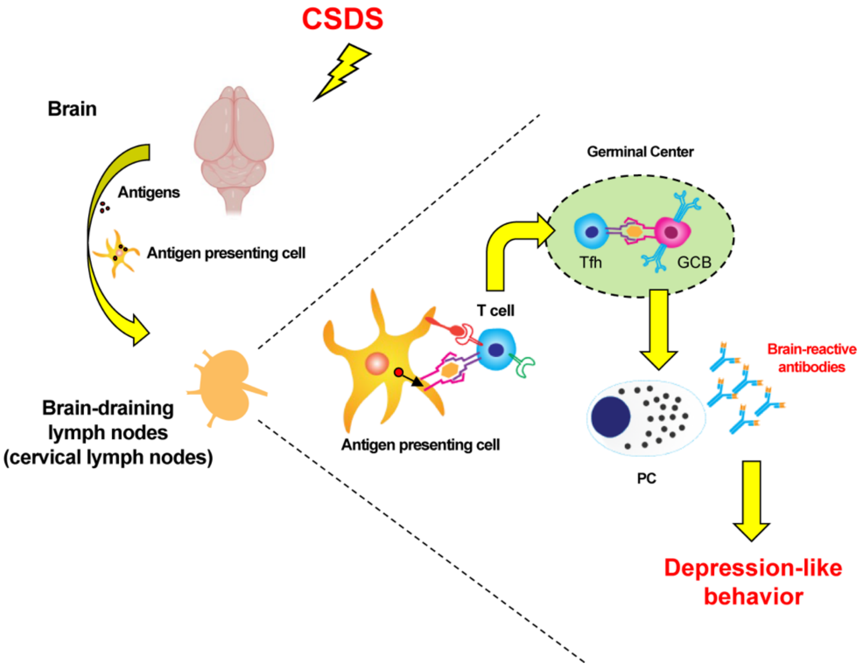

Fig. S8. Mechanisms of chronic social defeat stress (CSDS)-induced depression-like behavior mediated by production of autoantibodies against the brain. CSDS induces activation of the germinal center reaction preferentially in brain-draining lymph nodes, leading to production of brain-reactive antibodies. Increases in immune cell populations, such as germinal center B cells (GCB), follicular helper T cells (Tfh), and plasma cells (PC) in cervical lymph nodes are associated with low sociability in stressed mice. Furthermore, elevation of IgG antibody concentrations in the brain and brain-reactive antibody concentrations in sera correlate with depression-like behavior, which was dependent on the presence of B cells.

Table S1. List of antibodies used in this study.

Antibodies	Clone	Label	Supplier	Cat. No.	Dilution	Application
CD16/32	2.4G2	-	Bio X Cell	BE0307	1:200	FCM
TCRβ	H57-597	PerCPCy5.5	eBioscience	45-5961-80	1:400	FCM
PD1	J43	PECF594	BD Bioscience	562523	1:400	FCM
CXCR5	L138D7	BV421	Biolegend	145511	1:200	FCM
CD19	6D5	BV510	Biolegend	115545	1:200	FCM
CD19	6D5	Alexa700	Biolegend	115527	1:200	FCM
CD44	IM7	APC	BD Bioscience	561862	1:400	FCM
CD62L	MEL-14	FITC	BD Bioscience	553150	1:400	FCM
CD4	RM4-5	PECy7	BD Bioscience	561099	1:400	FCM
GL7	GL7	PE	Biolegend	144607	1:200	FCM
FAS	Jo2	BUV395	BD Bioscience	740254	1:200	FCM
CD138	281-2	BV605	BD Bioscience	563147	1:200	FCM
Foxp3	Fjk-16s	Alexa700	eBioscience	56-5773-82	1:200	FCM
CD31	MEC13.3	-	Biolegend	102501	1:400	IHC
GFAP	polyclonal	-	Dako	IS524	1:1	IHC
Donkey Anti-Mouse IgG (H+L)	polyclonal	Cy2	Jackson ImmunoResearch	715-225-150	1:300	IHC
Donkey Anti-Mouse IgG (H+L)	polyclonal	Cy3	Jackson ImmunoResearch	715-165-150	1:300	IHC
Donkey Anti-Rabbit IgG (H+L)	polyclonal	Cy5	Jackson ImmunoResearch	711-175-152	1:300	IHC
Donkey Anti-Rat IgG (H+L)	polyclonal	Cy2	Jackson ImmunoResearch	712-225-153	1:300	IHC
Donkey Anti-Rat IgG (H+L)	polyclonal	Cy3	Jackson ImmunoResearch	712-165-153	1:300	IHC
Donkey Anti-Goat IgG (H+L)	polyclonal	Cy5	Jackson ImmunoResearch	705-175-147	1:300	IHC
Purified Rat IgG2b, κ Isotype Ctrl Antibody	RTK4530	-	Biolegend	400671	-	B cell depletion (Control)
Purified anti-mouse CD20 Antibody	SA271G2	-	Biolegend	152104	-	B cell depletion
Donkey anti-Mouse IgG (H+L) Secondary Antibody	polyclonal	IRDye® 800CW	LI-COR	926-32212	1:5000	Western blotting

Abbreviations: FCM: Flow cytometry, IHC: Immunohistochemistry

Table S2. List of reagents used in this study.

Reagents	Supplier	Cat. No.	Application
BD Pharm Lyse	BD Bioscience	555899	Red blood cell lysis
Fixable Viability Dye eFluor®780	eBioscience	65-0865-14	FCM
True-Nuclear™ Transcription Factor Buffer Set	Biolegend	424401	FCM
Super Bright Complete Staining Buffer	eBioscience	SB-4401-42	FCM
DPBS	gibco	14190-144	FCM
UltraPure™ 0.5M EDTA	gibco	15575-038	FCM
Bovine Serum Albumin	sigma	A9647-100G	FCM
DAPI	molecular probes	D1306	IHC
Normal Donkey Serum	Jackson Immunoresearch	017-000-121	IHC
DEPEX Mounting medium	Electron Microscopy Sciences	13514	IHC
TritonX-100	sigma	T8787-100ML	IHC
20% Paraformaldehyde (formaldehyde) aqueous solution	Electron Microscopy Sciences	15713-S	IHC
1XTBS	Fisher	BP24721	Brain lysate preparation
cOmplete™, Mini, EDTA-free Protease Inhibitor Cocktail	Roche	11836170001	Brain lysate preparation
BCA Protein Assay Kit	Pierce	23227	Brain lysate preparation
IgG (Total) Mouse Uncoated ELISA Kit with Plates	Thermo	88-50400-22	ELISA
IgG (Total) Human Uncoated ELISA Kit with Plates	Thermo	88-50550-22	ELISA
ELISA Coating Buffer (5X)	Biolegend	421701	ELISA
Tween-20	sigma	P9416	Western blotting
Blocking Buffer for Fluorescent Western Blotting	ROCKLAND	MB-070	Western blotting
3X Blue Loading Buffer	Cell Signaling Technology	56036	Western blotting
30X Reducing Agent (1.25M DTT)	Cell Signaling Technology	14265	Western blotting

Abbreviations: FCM: Flow cytometry, IHC: Immunohistochemistry, ELISA: Enzyme-linked immunosorbent assay
